# Multimorbidity Trajectory Classes as Predicted by Race, Ethnicity, and Social Relationship Quality

**DOI:** 10.1093/geroni/igab046.3160

**Published:** 2021-12-17

**Authors:** Jason Newsom, AnneMarie O'Neill, Emily Denning, Ana Quiñones, Anda Botoseneanu, Heather Allore, Corey Nagel, David Dorr

**Affiliations:** 1 Portland State University, Portland, Oregon, United States; 2 Oregon Health & Science University, Portland, Oregon, United States; 3 University of Michigan - Dearborn, Dearborn, Michigan, United States; 4 Yale School of Medicine, New Haven, Connecticut, United States; 5 University of Arkansas for Medical Sciences, Little Rock, Arkansas, United States

## Abstract

Growth mixture modeling was used to classify multimorbidity (≥2 chronic conditions) trajectories over a 10-year period (2006-2016) in the Health and Retirement Study (N = 7,151, mean age = 68.6 years). Race/ethnicity (non-Hispanic Black, Hispanic, non-Hispanic White) and social relationship quality (positive social support and negative social exchanges, such as criticisms) were then used to predict trajectory class membership, controlling for age, sex, education, and wealth. We identified three trajectory classes: initial low levels and rapid accumulation of multimorbidity (increasing: 12.6%), initial high levels and gradual accumulation of multimorbidity (high: 19.5%), and initial low levels and gradual accumulation of multimorbidity (low: 67.9%). Blacks were more than twice as likely to be in the increasing (OR = 2.04, CI[1.29,3.21]) and high (OR = 2.28 CI[1.58,3.206]) multimorbidity groups compared with Whites, but there were no significant differences between Hispanics and Whites for either trajectory class (OR = .84 CI[.47,1.51]and OR = .74 CI[.41,1.34], respectively). Increments in perceived support were associated with significantly lower risk of membership in the increasing (OR = .59, CI[.46,.78]) and high classes (OR = .54 CI[.42,.69]), and increments in negative exchanges were associated with significantly higher risk of membership in the increasing (OR = 1.64 CI[1.19,2.25]) and high classes (OR = 2.22 CI[1.64,3.00]). These results provide important new information for understanding health disparities and the role of social relationships associated with multimorbidity in middle and later life that may aid in identifying those most at risk and suggesting possible interventions for mitigating that risk.

